# The Usefulness of Telemedicine in Perinatal Mental Health Services During and after COVID-19 Pandemic. Detailed Experience of the Team of „Together” Baby-Mother-Father Unit in Budapest

**DOI:** 10.1192/j.eurpsy.2022.79

**Published:** 2022-09-01

**Authors:** T. Kurimay, T. Fenyves, J. Szederkényi, G. Mező, A. Pelikán

**Affiliations:** North Centre Buda New Saint John Hospital and Oupatient Clinic, Buda Family Centre Mh Centre Department Of Psychiatry, Teaching Dep. Of Semmelweis University, Budapest, Hungary

**Keywords:** Telementalhealth, Usefullness of e-platform for perinatal women, Effective tool during Pandemic, Baby-Mother-Father programme

## Abstract

Due to the nature of the perinatal period, it affects generations who are more at home in the electronic space, hence some form of telemedicine can be used in a number of areas. The “Together” Baby-Mother-Father Integrated Program has been running since 2004. Both the condition of those affected and the current epidemic makes it difficult for patients and their families to access adequate perinatal specialist care. At the beginning of the epidemic, the switch to telemedicinal psychiatric care has been rapid and focused mainly on the use of Phone, Skype, Viber and Email. To our findings the advantages include, easier access to care, and more frequent contacts. The home environment is accessible and the families are more involved. Also, care does not compete with the scarce resources of time and space. Some of the possible disadvantages are, that more work on intimacy is needed, and the treatability of certain diseases is questionable (e.g., psychoses). Care is less documentable with the current regulations, and funding has not yet been adapted to the changes. The telemedicinal care and support network in Hungary - among many - contains an online medical system (EESZT) including e-prescription. Online- psychotherapy, consultation, peer-group platforms. There is a non-stop hotline for patients, etc. In 2021 the total number of our cases increased by 34%, but realistically the visit number was also higher, due to the amount of shorter telephone and e-mail interactions. Depression and bipolar disorder were among the highest proportion by the patients present.

**Disclosure:**

No significant relationships.

